# Comparative assessment of methods for estimating individual genome-wide homozygosity-by-descent from human genomic data

**DOI:** 10.1186/1471-2164-11-139

**Published:** 2010-02-25

**Authors:** Ozren Polašek, Caroline Hayward, Celine Bellenguez, Veronique Vitart, Ivana Kolčić, Ruth McQuillan, Vanja Saftić, Ulf Gyllensten, James F Wilson, Igor Rudan, Alan F Wright, Harry Campbell, Anne-Louise Leutenegger

**Affiliations:** 1 Public Health Sciences, University of Edinburgh, Edinburgh, UK; 2 Department of Medical Statistics, Epidemiology and Medical Informatics, Andrija Stampar School of Public Health, Medical School, University of Zagreb, Zagreb, Croatia; 3 Human Genetics Unit, Medical Research Council, Edinburgh, UK; 4 Inserm, U535, Villejuif, France; 5 University Paris-Sud, IFR69, UMR_S535, Villejuif, France; 6 University Hospital Sestre Milosrdnice, Zagreb, Croatia; 7 Department of Genetics and Pathology, Rudbeck Laboratory, Uppsala, Sweden; 8 Gen-info d.o.o., Zagreb, Croatia; 9 Croatian Centre for Global Health, University of Split, Split, Croatia

## Abstract

**Background:**

Genome-wide homozygosity estimation from genomic data is becoming an increasingly interesting research topic. The aim of this study was to compare different methods for estimating individual homozygosity-by-descent based on the information from human genome-wide scans rather than genealogies. We considered the four most commonly used methods and investigated their applicability to single-nucleotide polymorphism (SNP) data in both a simulation study and by using the human genotyped data. A total of 986 inhabitants from the isolated Island of Vis, Croatia (where inbreeding is present, but no pedigree-based inbreeding was observed at the level of F > 0.0625) were included in this study. All individuals were genotyped with the Illumina HumanHap300 array with 317,503 SNP markers.

**Results:**

Simulation data suggested that multi-point FEstim is the method most strongly correlated to true homozygosity-by-descent. Correlation coefficients between the homozygosity-by-descent estimates were high but only for inbred individuals, with nearly absolute correlation between single-point measures.

**Conclusions:**

Deciding who is really inbred is a methodological challenge where multi-point approaches can be very helpful once the set of SNP markers is filtered to remove linkage disequilibrium. The use of several different methodological approaches and hence different homozygosity measures can help to distinguish between homozygosity-by-state and homozygosity-by-descent in studies investigating the effects of genomic autozygosity on human health.

## Background

A number of studies in plants and animals have suggested that an increased level of genome-wide homozygosity is expected to have negative effects on health, fitness and survival in a wide range of environmental conditions [1-3]. These studies were commonly performed using a small number of genetic markers [4], and consequently were underpowered to detect true effects. The overall conclusions were also sensitive to upward publication bias [5,6].

In contrast, studies on the effects of homozygosity levels on human biology and health are quite rare. One of the first accounts of the beneficial effects of heterozygosity was proposed by Penrose, who suggested that increased heterozygosity may have beneficial effects on a large number of human traits [7]. Subsequent research was mainly focused mainly on the effects of inbreeding on human fertility, early morbidity and mortality, and the effects on quantitative biological traits [8-10]. Estimates of inbreeding coefficients in human studies have traditionally been computed from genealogical data, although their reliability has often been in question, with problems including incomplete genealogical records or false paternities [11]. A more recent approach used biological markers to estimate homozygosity, ranging historically from blood groups [12] to more recent DNA-based markers [13-15]. DNA markers have recently become a powerful tool to measure individual genome-wide homozygosity and two different marker types can be used, each with its strengths and weaknesses - short tandem repeats (STR) and single-nucleotide polymorphisms (SNP). The former are considerably more informative, but the latter are far more numerous across the human genome and with the advent of array-based typing technologies also economically more feasible to determine on a genome wide scale.

Several different methods for the estimation of individual genome-wide homozygosity have been developed in both animal and human genetics. The most basic measure (actually a measure of heterozygosity rather than homozygosity) is multilocus heterozygosity, defined as the proportion of heterozygous loci in all genotyped loci of an individual [16]. Internal relatedness (IR) or allelic distance [6] are commonly used in animal genetics, but neither of these have been used in human genetics studies. Three additional methods have recently been developed in human genetics recently in order to estimate the inbreeding coefficients from genetic marker data, thus avoiding reliance on genealogy data [13-15,17]. The first approach [13] employs multi-point information (i.e., using marker dependencies via a hidden Markov model) and has been successfully used in several homozygosity mapping studies where it helped to map a locus responsible for e.g. Taybi-Linder syndrome [18]. The second method [14] is a single-point approach (i.e. not using marker dependencies) and has been shown to correlate well with theoretical expectations from population demography and genetic structure [14,19]. The estimates based on this approach have been reported in one study to correlate well with several health-related quantitative traits [20]. The last approach [21] is also a single-point method but has not been evaluated yet.

The aim of this study was to compare and validate the results obtained by four different methods to estimate individual homozygosity-by-descent (HBD) using human genome-wide SNP scan data from a Croatian isolated population.

## Methods

### Subjects

This study was based on data obtained from a large genetic epidemiology project that is being carried out in the isolated islands of Croatia. The initial goals of this project were to describe and understand human variation by investigating isolated communities [22,24], and to investigate the effects of inbreeding in those communities [20,25]. Subsequent efforts have been oriented towards understanding the genetic background of complex human traits and diseases.

A total of 986 inhabitants of the Croatian Island of Vis were included in this study. The population on the island of Vis has been well characterized in terms of demographic and population genetic events [23], and these have suggested that the population has experienced several bottleneck events in the relatively recent past, within the last 25 years. All examinees were over 18 and signed informed consent before entering the study. The study has been approved by the relevant Ethical Committees in both Scotland and Croatia.

**Figure 1:**
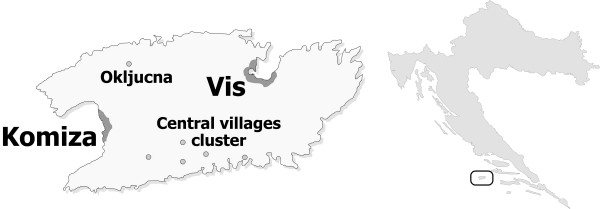
**Geographical position and settlements on the Vis Island, Croatia**.

Genealogical information for examinees was available for 3-4 ancestral generations in nearly all cases (and in some cases up to 6 generations), based on the self-reported information and parish records. No inbreeding loops suggestive of a parental relationship of first-cousin (F = 0.0625) or closer were seen in the genealogical data, confirming the strong influence of the local Catholic Church on the avoidance of inbreeding [26]. Despite this, cryptic inbreeding was still expected to be found due to the known effect of limited mate choice in isolated populations [2]. All individuals included in this study were classified into seven groups of grandparental birthplace cluster, based on *a-priori *expectations of expected genome-wide homozygosity levels. This was based on a combination of information from genealogical and demographic sources (Table 1). The highest homozygosity estimates were expected in the village of Okljucna which is a small and isolated outback settlement on the island. Secondly, Komiza is a larger village which is also isolated, but historically experienced more immigration than Okljucna. The third group included examinees from villages in the central highlands. The fourth group consisted of examinees all four of whose grandparents originated from the village of Vis, which historically had more connections with the mainland. The fifth group consisted of individuals of mixed origin (where at least one grandparent was from the island). Finally, the last two groups consisted of examinees all four of whose grandparents originated from the rest of Croatia, or even from other countries (Figure 1).

**Table 1:** Grandparental birthplace cluster of examinees in seven groups with progressively reduced expected individual genome-wide homozygosity (after removal of 63 individuals due to missing genotypes of over 5%)

Group	Description	N	%
I	All four grandparents from Okljucna	17	1.8
II	All four grandparents from Komiza	244	26.4
III	All four grandparents from the central villages	68	7.4
IV	All four grandparents from Vis	115	12.5
V	Mixed origin (at least one grandparent from the island)	229	24.8
VI	All four grandparents from the rest of Croatia	200	21.7
VII	All four grandparents from the other countries	50	5.4

Total		923	100.0

### Genotyping

DNA was obtained from blood samples provided by all examinees, which were frozen on the site and then sent to the lab for DNA extraction. Extraction was performed using Nucleon kits (Tepnel, UK), at the Institute for Anthropological Research in Zagreb, Croatia. A total of 986 individuals were genotyped using the Illumina HumanHap300 (v1) array, with a total of 317,503 SNP markers.

We excluded 63 individuals because of genotyping rate lower than 95%. We then removed 864 markers based on departure from Hardy-Weinberg equilibrium (P ≤ 10E-07; we used a small threshold because we only wanted to identify very ill behaved markers and did not expect HWE in an isolated population), 17,856 markers due to low call rate (<95%) and 10,552 markers due to low minor allele frequency (MAF < 0.05). Additionally, only the autosomal markers for which a genetic location was available from Illumina were included. This left a total of 274,577 SNPs and 923 samples. The quality control procedure was performed with PLINK, version 1.01 [available from http://pngu.mgh.harvard.edu/purcell/plink/].

### Homozygosity and homozygosity-by-descent estimation

Five different measures were compared:

#### Multilocus heterozygosity (MLH)

This is the proportion of heterozygous loci [27], equivalent to one minus genome-wide homozygosity.

#### Expected genome-wide homozygosity (F_PLINK_) and locus-based homozygosity (F_ADC_

These two methods use genome-wide marker genotypes, but the information from each marker is used independently of the others. We hence refer to these approaches as single-point methods. F_ADC _was initially described using microsatellite markers [14] and F_PLINK _using SNPs [21]. F_PLINK _relies on genome-wide expected homozygosity, while F_ADC _is based on the summation of locus-based homozygosity information. In this study the weighted approach for F_ADC _was used, in which estimates are weighted by the inverse of their variance in order to obtain more precise estimates [14]. For F_PLINK_, we used the '--het' command in PLINK version 1.01.

#### Maximum likelihood approaches: single-point (FEstimSPT) and multi-point (FEstim)

FEstim is a maximum likelihood approach that estimates the genome-based inbreeding coefficient of an individual [13]. Marker dependencies are taken into account through the use of a hidden Markov model. This modelling allows long homozygous stretches to contribute strongly to the inbreeding estimation while isolated homozygous markers will tend to be ignored. In addition, the presence of rare alleles in a homozygous stretch will help boosting its contribution to inbreeding.

For comparison purposes, we also computed a single-point version of FEstim (referred to as FEstimSPT). At each marker locus, it uses the same modelling as FEstim but ignores marker dependencies (i.e. the hidden Markov structure). FEstim version 1.2 was used for computations [available upon request: anne-louise.leutenegger@inserm.fr].

The multi-point approach, FEstim assumes linkage equilibrium and may provide inflated inbreeding coefficient estimates if this assumption is violated [13]. Haplotypes that are indeed frequent because of LD will not be taken into account properly by the method and will tend to wrongly provide increased evidence for HBD. In order to select SNP markers we used MASEL [28] to remove linkage disequilibrium (LD) present among SNPs, and then applied the FEstim calculation. MASEL selects a set of markers based on LD while maximizing for marker information content, genome coverage and number of selected markers (set size) [available upon request: celine.bellenguez@inserm.fr]. MASEL has been applied in the framework of linkage analysis but the issues in terms of LD are the same here. We considered two different LD thresholds: r^2 ^≤ 0.1 (M0.1) which selected a set of 49,987 SNPs (~18% of the original SNP number) and r^2 ^≤ 0.05 (M0.05) which selected 16,339 SNPs (~6%). LD was estimated from HapMap CEU data.

### Simulation information

In order to investigate the correlation between some of these methods, we simulated genotypes for the offspring of first cousins, second cousins and third cousins by gene-dropping on the genealogy (Genedrop program of MORGAN2.7 [available from the Pangaea Web site, http://www.stat.washington.edu/thompson/Genepi/pangaea.shtml]). We used the marker map from a 10K Affymetrix chip with linkage disequilibrium removed, leaving a total of 4,849 SNPs in the analysis dataset. A total of 10,000 replicates were performed. Because the data were simulated, we could determine which loci were homozygous-by-descent (HBD) and which were not. This allowed us to compute the true proportion of loci that were HBD and hence the true inbreeding coefficient of an individual. For comparison purposes, the negative values reported by F_PLINK _and F_ADC _were set to zero.

### Statistical analysis

The Spearman rank test was used for the calculation of correlation coefficients, while the Mann-Whitney test was used for significance testing between two of the groups, including pair-wise comparisons of the neighbouring clusters. Wilcoxon's test was used to analyze homozygosity estimates between siblings. Statistical analyses were performed in SPSS ver. 13 (SPSS Inc., Chicago, IL), with the threshold for statistical significance set at P < 0.05.

## Results

Simulation results in the three different scenarios (offspring of first, second or third cousins) yielded the highest correlation coefficients of true homozygosity-by-descent with FEstim; correlation coefficients of true HBD for the single-point methods (F_PLINK_, F_ADC _and FEstimSPT) were comparable to one another, but always lower than that of FEstim (Table 2). The single-point approaches seemed to show worsening of the correlations with the true HBD and larger mean estimates compared to the truth in the situations with lower inbreeding coefficients (offspring of second and third cousins). This suggests that these methods will tend to yield inflated estimates in populations with low inbreeding coefficients, most likely due to isolated homozygous SNPs (Table 2).

**Table 2:** Simulation results for offspring of first cousins (1C), second cousins (2C) and third cousins (3C).

			Correlation coefficients				
			
		Mean [95% CI]	True HBD	F_PLINK_	F_ADC_	FEstimSPT	FEstim
1C	True HBD	0.062 [0.019-0.121]	1.00	0.82	0.82	0.86	0.91
	F_PLINK_	0.063 [0.005-0.129]	0.82	1.00	1.00	0.95	0.78
	F_ADC_	0.063 [0.006-0.129]	0.82	1.00	1.00	0.95	0.78
	FEstimSPT	0.063 [0.004-0.128]	0.86	0.95	0.95	1.00	0.82
	FEstim	0.063 [0.020-0.115]	0.91	0.78	0.78	0.82	1.00
2C	True HBD	0.015 [0.000-0.045]	1.00	0.51	0.51	0.56	0.87
	F_PLINK_	0.018 [0.000-0.059]	0.51	1.00	0.99	0.82	0.52
	F_ADC_	0.018 [0.000-0.059]	0.51	0.99	1.00	0.83	0.52
	FEstimSPT	0.016 [0.000-0.055]	0.56	0.82	0.83	1.00	0.58
	FEstim	0.016 [0.000-0.045]	0.87	0.52	0.52	0.58	1.00
3C	True HBD	0.004 [0.000-0.020]	1.00	0.22	0.22	0.26	0.77
	F_PLINK_	0.009 [0.000-0.041]	0.22	1.00	0.97	0.70	0.25
	F_ADC_	0.009 [0.000-0.040]	0.22	0.97	1.00	0.71	0.26
	FEstimSPT	0.007 [0.000-0.035]	0.26	0.70	0.71	1.00	0.30
	FEstim	0.004 [0.000-0.023]	0.77	0.25	0.26	0.30	1.00

All F_PLINK _and F_ADC _values that were negative were set to zero in these correlations, in order to allow comparisons across different methods since FEstim does not provide negative values.

Correlation coefficient is for comparing each method to the true proportion of markers that are homozygous by descent ("True HBD" line).

On the Vis island dataset, the mean heterozygosity for the entire sample and full marker set was 0.354, suggesting that 35.4% of genotyped SNP markers in full marker count were heterozygous and 64.6% homozygous (Table 3). Interestingly, estimates of the single-point approaches (F_PLINK_, F_ADC _and FEstimSPT) were not substantially affected by changes in marker selection, while both MLH and FEstim were. Notably, the average value of FEstim calculated with the less restrictive MASEL selection threshold (M0.1) was twice greater than the more restrictive selection, illustrating the inflation in inbreeding estimates in the presence of LD using multi-point approaches. As for the MLH estimates, their values increased with marker selection sets since MASEL always selects the most informative markers (highest heterozygosity). There were no substantial differences in the standard errors or in the ranges between the same methods, when used with different thresholds for LD.

**Table 3:** Descriptive statistics of various homozygosity estimates and marker sets in the Vis Island dataset

Method	Mean	St. deviation	Range	Minimum	Maximum
MLH	0.354	0.005	0.040	0.325	0.365
MLH, M0.1	0.390	0.006	0.044	0.360	0.405
MLH, M0.05	0.360	0.005	0.042	0.329	0.371
F_PLINK_	0.009	0.014	0.116	-0.021	0.094
F_PLINK_, M0.1	0.009	0.014	0.112	-0.021	0.090
F_PLINK_, M0.05	0.009	0.016	0.110	-0.026	0.084
F_ADC_	0.009	0.015	0.112	-0.021	0.091
F_ADC_, M0.1	0.009	0.014	0.113	-0.023	0.083
F_ADC_, M0.05	0.008	0.016	0.109	-0.026	0.083
FEstimSPT	0.007	0.011	0.083	0.000^a^	0.083
FEstimSPT, M0.1	0.008	0.012	0.079	0.000^a^	0.079
FEstimSPT, M0.05	0.008	0.011	0.084	0.000^a^	0.084
FEstim, M0.1	0.017	0.010	0.086	0.000^a^	0.086
FEstim, M0.05	0.009	0.011	0.080	0.000^a^	0.080

^a ^By construction in the methods, all estimates will be between zero and one.

**Figure 2:**
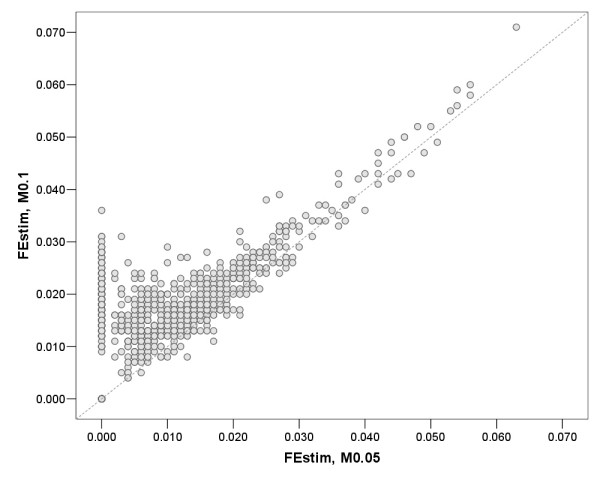
**Scatterplot of FEstim using two marker selections, M0.1 and M0.05**. Dashed line is a reference line (y = x).

The correlation between methods was high (Table 4). Single-point approaches were almost completely correlated with MLH, whatever the marker selections were used. Correlation coefficients between multi-point and other measures were lower, with correlation coefficient mostly in the range of 0.50-0.60. A comparison of genome-wide homozygosity estimates between siblings (a total of 117 sibling pairs were identified in the entire sample) revealed that none of the methods showed statistically significant different estimates within sibling pairs (analysis performed in pair-wise fashion using Wilcoxon test; data not shown). The FEstim estimates for the two marker selections (M0.1 and M0.05) showed relatively low correlation (0.51). However, detailed analysis revealed that the correlation for the examinees who were classified as inbred was much higher (when all examinees with FEstim-M0.05 value of zero are removed), yielding a corrected correlation coefficient of 0.74 (Figure 2). Similarly, the corrected correlation coefficients of FEstim with each of the single-point methods (for marker selection M0.05) ranged between 0.60-0.67, suggesting that correlation coefficients for various homozygosity methods will be higher in more inbred individuals.

**Table 4:** Correlation coefficients between the five methods in three marker selection sets in the Vis Island dataset

		MLH	F_PLINK_	F_ADC_	FEstimSPT	FEstim
Full marker set	MLH	1.00	-1.00	-1.00	-0.67	*
	F_PLINK_	-1.00	1.00	0.99	0.67	*
	F_ADC_	-1.00	0.99	1.00	0.66	*
	FEstimSPT	-0.67	0.67	0.66	1.00	*
	FEstim	*	*	*	*	*
M0.1	MLH	1.00	-1.00	-0.99	-0.71	-0.54
	F_PLINK_	-1.00	1.00	0.99	0.74	0.60
	F_ADC_	-0.99	0.99	1.00	0.78	0.63
	FEstimSPT	-0.71	0.74	0.78	1.00	0.55
	FEstim	-0.54	0.60	0.63	0.55	1.00
M0.05	MLH	1.00	-1.00	-0.99	-0.73	-0.64
	F_PLINK_	-1.00	1.00	0.99	0.73	0.64
	F_ADC_	-0.99	0.99	1.00	0.75	0.64
	FEstimSPT	-0.73	0.73	0.75	1.00	0.51
	FEstim	-0.64	0.64	0.64	0.51	1.00

All correlations were significant at the level of P < 0.001

All F_PLINK _and F_ADC _values that were negative were set to zero in these correlations, in order to allow comparisons across different methods since FEstim does not provide negative values

*FEstim was not calculated for the full marker set due to LD

**Figure 3:**
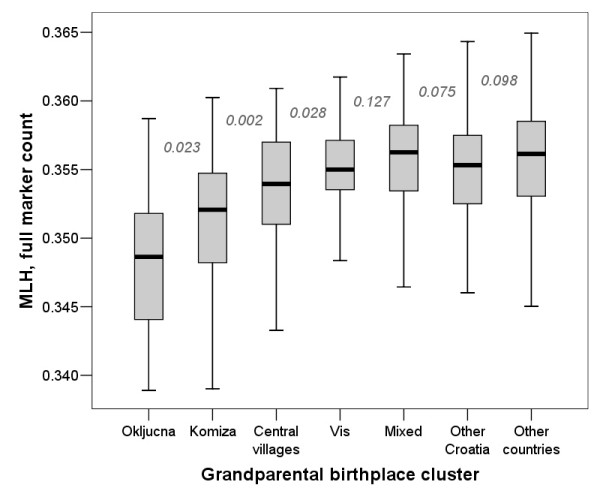
**Grandparental birthplace clusters and their homozygosity estimates using MLH (full marker count)**. Numbers on the figure are P values of pair-wise comparisons between neighbouring group homozygosity estimates using Mann-Whitney test.

**Figure 4:**
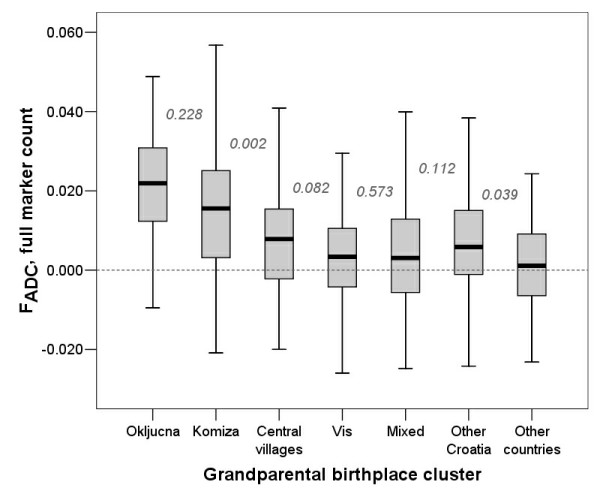
**Grandparental birthplace clusters and their homozygosity estimates using F_ADC _(full marker count)**. Numbers on the figure are P values of pair-wise comparisons between neighbouring group homozygosity estimates using Mann-Whitney test. Plot for F_PLINK _is not shown here due to very high correlation coefficient with F_ADC_.

**Figure 5:**
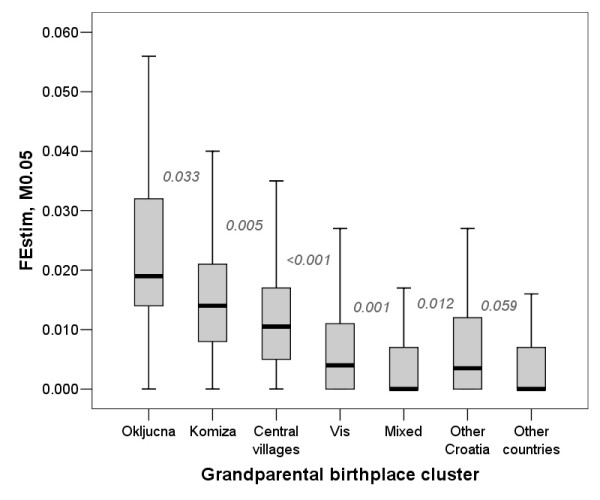
**Grandparental birthplace clusters and their homozygosity estimates using FEstim (M0.05 selection)**. Numbers on the figure are P values of pair-wise comparisons between neighbouring group homozygosity estimates using Mann-Whitney test.

Analysis of the homozygosity estimates in relation to grandparental birthplace cluster showed gradually decreasing homozygosity estimates with decrease in the expected degree of isolation (Figure 3). Interestingly, it highlighted a difference between FEstim and the other methods in terms of estimating homozygosity in mixed individuals and those coming from the rest of Croatia. The difference between groups V and VI (mixed vs. other Croatia) was not statistically significant for MLH (Figure 3) or F_ADC _(Figure 4), while FEstim (M0.05) estimates were statistically significantly different between these two groups, possibly detecting cryptic inbreeding in the group in which some examinees had all four grandparents from a single village somewhere in Croatia, other than the island of Vis (Figure 5). It is also worth noticing that MLH only managed to significantly differentiate between the first four neighbouring clusters, F_ADC _managed to differentiate only two neighbouring clusters, while FEstim managed to significantly differentiate all but one clusters (Figure 5).

## Discussion

The abundance of genome-wide homozygosity methods available today presents an interesting challenge for researchers. The choice of method may affect the results, and it is therefore important to understand the characteristics of each method. The main finding of this study is that different genome-wide homozygosity methods are sensitive to different parameters, and may be more or less suited to various study designs. MLH is a robust method but provides homozygosity-by-state (HBS) information only and is therefore of limited use in inbreeding and admixture studies as has been previously reported [5,6,14]. The group of single-point approaches (F_PLINK_, F_ADC _and FEstimSPT) are highly correlated to one another, and also highly correlated to MLH (especially for the first two). The multi-point method FEstim, which takes neighbouring marker information into account, was the only method that managed to clearly differentiate between groups of various degrees of endogamy. This is in agreement with the fact that multi-point approaches should provide more HBD information, as suggested by the simulation study. In the presence of linkage disequilibrium, one has to use some care in applying multi-point methods, by either removing markers in LD (as we did with MASEL) or by including LD in the data modelling. The results of this study suggest that even a small amount of LD may affect the results of multipoint homozygosity methods, as seen in the difference between FEstim measures that were based on the MASEL 0.1 and 0.05 cut-offs. The correlation between very restrictive and less restrictive marker selection suggested that inbreeding estimates in more inbred individuals will be similar, while in less inbred individuals the presence of LD will tend to overinflate inbreeding estimates (as seen in the comparison of FEstim M0.1 and FEstim M0.05 in the Figure 2). Single-point measures did not seem to be strongly affected by the presence of LD.

Results from some animal studies have suggested that molecular-marker based estimates may not be the optimal way of measuring genome-wide heterozygosity [29], as these may provide estimates that are different between siblings who are expected to have the same pedigree-based inbreeding coefficient. Here we have shown that, although estimates are different between sibs, none of the investigated methods suffered from significant sibling differences, suggesting that the use of large marker sets to boost statistical power may yield more precise estimates compared to studies that are based on a handful of markers.

One of the problems that may arise in individual genome-wide homozygosity estimation with methods that give more weight to rare alleles, is the introduction of foreign individuals into an isolated population, sometimes referred to as "sample contamination" [21]. This is due to the introduction of immigrant alleles, which can by a definition become "rare" for the island population. The main consequence of this is that methods that give more weight to rare alleles may overestimate the inbreeding coefficients of immigrants [30]. Our results do not seem to suffer from this bias, as we did not detect any indication of an overinflated inbreeding coefficient in the immigrants group, who likely have alleles that are "rare" in the isolated and endogamous island population. This issue might be a special problem in unequally mixed populations where it may be difficult to separate rare alleles of the isolated population from alleles brought in by immigrants.

Demographic history and population genetic structure may have a strong effect on individual genome-wide homozygosity values in a population. We observed a gradual decrease in average genome-wide homozygosity, which was in line with expectations based on demographic history and decreasing levels of endogamy, as previously reported [23]. The most endogamous village of Okljucna had the lowest MLH values and the highest F_ADC _and FEstim values (the large variation was the consequence of a small sample size of only 17 individuals). Other groups had decreasing homozygosity values, but notably FEstim managed to distinguish between groups V and VI, indicating that only this method is capable of detecting cryptic inbreeding (due to the fact that some individuals from group VI had originated from highly endogamous marriages, as their grandparents have originated from the same village elsewhere in Croatia, based on the available data provided by the examinees).

The shortcomings of this study include the low sample size for some groups (namely Okljucna, which consisted of 17 examinees only). Although humans generally experience lower inbreeding coefficients than many plant and animal species, it is very interesting to explore patterns of cryptic, more ancient inbreeding in humans, which may have strong effect on some human traits [19]. Another shortcoming is the fact that the simulation study was not done with very dense markers in high LD (but only with SNPs every 0.8 cM) which does not allow us to draw the most general conclusions from these simulations. Although this is true, we do believe that Vis island data provide strong information about the different methods. For instance, we do not feel that the single-point approaches will ever be able to extract proper HBD information from the marker data as can be seen from their high correlation to the MLH. In addition, even if there is still some LD left in M0.05 map (a SNP every 0.2 cM), we feel that the estimated inbreeding values do reflect better the HBD information from each individual as illustrated by the differentiation of the various endogamy groups (Figure 5). More intensive simulation studies (very dense SNP map and LD) are underway to confirm these results.

## Conclusions

This study provides the most comprehensive comparison of different genome-wide homozygosity measures to date. Our findings suggest that the most commonly used single-point methods (F_PLINK _and F_ACD_) do not measure much more than the simple proportion of heterozygous loci (multilocus heterozygosity), but they do have the advantage of not being sensitive to the presence of linkage disequilibrium. Multi-point FEstim is the best approach tested here for inbreeding estimation from genetic markers (i.e. the closest to the true HBD information) when there is no LD present. It remains unclear which is the best method when there are dense markers with high LD. The next substantial advancement in the study of genome-wide homozygosity levels is likely to be based on fully sequenced human genomes, providing an even more precise estimate of individual genome-wide homozygosity and its distribution across the genome.

## Authors' contributions

OP, ALL, HC, AW and IR conceived the study; CH and VV performed laboratory procedures and data cleaning; IR, OP, IK and VS performed the sampling and organization of field work; OP and ALL drafted the manuscript; ALL, CB and OP wrote the scripts for the homozygosity estimation; UG, JFW, RM and AW contributed to the idea and provided conceptual ideas for the initial draft broadening and adding the crucial study elements; OP and ALL performed the statistical analysis. All authors read and approved the final version of manuscript.
